# 

**Published:** 1880-10

**Authors:** 


### Table of Contents,

### ORIGINAL COMMUNICATIONS.

The Uses of Tar-Water in Obstetrical and Gynæcological Practice. By Joseph Eve Allen, M.D., Augusta, Ga. .......... 385
Comparative Influence of Certain Localities on Health, particularly in the production of Consumption. By H. P. Gatchell, M.D., Atlanta, Ga .......... 393

### REPORTS OF SOCIETIES.

The British Medical Association .......... 412

### SELECTIONS.

Typhoid Fever .......... 433

### EDITORIAL.

Electricity .......... 440
Meteorological Report for September .......... 443

### BIBLIOGRAPHICAL.

Treatise on Therapeutics .......... 444
System of Medicine .......... 444
A Treatise on Common Forms of Functional Nervous Diseases .......... 445
The Brain as an Organ of Mind .......... 445
Table of Tests and Solidities of the Alkaloids Appended .......... 445
Pomphlets Received .......... 445

### EXCERPTA.

Best Mode of giving Ergot .......... 446
Stigmata of Maize .......... 446
Transmission of Syphilis from the Father .......... 448

